# Random unstimulated pediatric luteinizing hormone levels are not reliable in the assessment of pubertal suppression during histrelin implant therapy

**DOI:** 10.1186/1687-9856-2013-20

**Published:** 2013-12-02

**Authors:** E Kirk Neely, Lawrence A Silverman, Mitchell E Geffner, Theodore M Danoff, Errol Gould, Paul S Thornton

**Affiliations:** 1 Pediatric Endocrinology and Diabetes, Stanford University, Stanford, California, USA; 2 Pediatric Endocrinology, Goryeb Children’s Hospital, Atlantic Health System, Morristown, New Jersey, USA; 3 Division of Endocrinology, Diabetes, and Metabolism, and The Saban Research Institute, Children’s Hospital Los Angeles, Los Angeles, California, USA; 4 Endo Pharmaceuticals Inc., Malvern, Pennsylvania, USA; 5 Department of Endocrinology, Cook Children’s Medical Center, Fort Worth, Texas, USA

**Keywords:** Central precocious puberty, Estradiol, GnRHa, Histrelin, Luteinizing hormone

## Abstract

**Background:**

Gonadotropin-releasing hormone agonist (GnRHa)-stimulated luteinizing hormone (LH) is the standard hormonal assessment for both diagnosis and therapeutic monitoring of children with central precocious puberty (CPP). Use of unstimulated (random) LH levels may be helpful in diagnosis and has gained popularity in monitoring GnRHa therapy despite lack of validation against stimulated values. The objective of this investigation was to assess the suitability of random LH for monitoring pubertal suppression during GnRHa treatment.

**Methods:**

Data from a multi-year, multicenter, open-label trial of annual histrelin implants for CPP was used for our analysis. Children meeting clinical and hormonal criteria for CPP, either naïve to GnRHa therapy or previously treated with another GnRHa for at least 6 months who were being treated at academic pediatric centers were included in the study. Subjects received a single 50-mg subcutaneous histrelin implant annually until final explant at an age determined at the discretion of each investigator. Monitoring visits for physical examination and GnRHa-stimulation testing were performed at regular intervals. The main outcome measure was pubertal suppression during treatment defined by peak LH < 4 mIU/mL after GnRHa stimulation.

**Results:**

During histrelin treatment, 36 children underwent a total of 308 monitoring GnRHa stimulation tests. Unstimulated and peak LH levels were positively correlated (r = 0.798), and both declined from the first to second year of treatment. Mean ± SD peak LH level during therapy was 0.62 ± 0.43 mIU/mL (range, 0.06–2.3), well below the normal prepubertal mean. Mean random LH was 0.35 ± 0.25 mIU/mL (range, 0.04–1.5), 10-fold higher than the normal prepubertal mean. The random LH levels were above the prepubertal upper threshold (<0.3 mIU/mL) in 48.4% of all tests and in 88.9% of subjects at some point during therapy.

**Conclusions:**

In contrast with GnRHa-stimulated LH, unstimulated LH values frequently fail to demonstrate suppression to prepubertal values during GnRHa therapy for CPP, despite otherwise apparent pubertal suppression, and are thus unsuitable for therapeutic monitoring.

**Trial registration:**

ClinicalTrial.gov NCT00779103.

## Background

Gonadotropin-releasing hormone agonist (GnRHa) therapy is considered the standard of care for treatment of children with central precocious puberty (CPP) [1]. Monthly and 3-monthly depot leuprolide [2-5] and the annual histrelin implant [6,7] are the most commonly used therapies in the U.S. Serum LH response to GnRH or GnRHa stimulation, typically using aqueous leuprolide acetate as the stimulating agent, is the conventional test used to diagnose CPP [8-15] in conjunction with sex steroid levels and characteristic clinical features. Unstimulated, i.e. random, LH levels can also be used for diagnosis of CPP, although with limited sensitivity in early puberty [16].

During treatment of CPP, GnRHa stimulation tests are utilized to monitor and confirm continued LH suppression, with peak levels dropping into or below the normal prepubertal range [2-7,17,18]. During depot GnRHa therapy, stimulation testing can be performed easily by drawing a post-injection sample, whereas monitoring during histrelin implant therapy requires an additional injection. Many practitioners alternatively use a random LH level during therapeutic monitoring instead of stimulation testing, despite the lack of data demonstrating its utility. Here we investigate the suitability of random LH levels for assessment of pubertal suppression during histrelin implant therapy using data collected from the pivotal, long-term, U.S. multicenter trial of histrelin for treatment of CPP [6,7].

## Methods

Data were derived from an open-label study of 36 children with CPP [6,7] in which all subjects received a 50-mg subcutaneous histrelin implant (Supprelin LA, Endo Pharmaceuticals, Malvern, PA) in the upper arm annually until final explant at an age determined at the discretion of each investigator. The study was open to girls aged 2–8.99 years and to boys aged 2–9.99 years who had evidence of CPP and who had not previously received GnRHa therapy (“treatment naïve”) and to girls aged 2–10.99 years and boys aged 2–11.99 years who had received treatment with a GnRHa regimen for at least 6 months (“previously treated”). Prior to any treatment, all participants had breast Tanner stage ≥ 2 (girls) or testicular volume ≥ 4 mL (boys), stimulated LH > 7 mIU/mL after GnRH or > 10 mIU/mL after leuprolide acetate, and bone age ≥ +2 standard deviations [SD]. Written informed consent was obtained at each site, as was assent when appropriate.

Monitoring visits with physical examinations and hormone testing were performed at months 1, 3, 6, 9, 12, 13, 18, 24, 36, and every 6 months thereafter. Blood samples were collected at 0 minutes (baseline) before subcutaneous injection of leuprolide acetate (20 μg/kg) and 30 and 60 minutes post-injection; beginning in the third year of the study, samples were obtained at 0 and 40 minutes. All hormone measurements were performed at Esoterix Clinical Trial Services (East Windsor, NJ). LH and follicle-stimulating hormone (FSH) were measured by immunochemiluminescent (ICMA) assay with a lower limit of quantification of 0.02 mIU/mL [15]. Mean values are stated with standard deviations. Estradiol was initially measured on baseline samples using radioimmunoassay (RIA) with a lower limit of detection of 5 pg/mL, but, beginning in the third year of the study, assays were performed by liquid chromatography and tandem mass spectrometry (LCMS/MS) with a lower limit of 1 pg/mL. For consistency of data analysis, any estradiol value ≤ 5 pg/mL was imputed as 5 pg/mL. Testosterone measurement was performed on baseline samples using RIA with a lower limit of 3 ng/dL.

## Results

Mean age at the onset of GnRHa therapy was 7.1 ± 1.4 years in the treatment-naïve group (n = 20) and 8.9 ±1.5 years in the previously treated group (n = 16). Mean ± SD unstimulated (0 minute) and peak stimulated (30 or 60 minute) LH levels on the day of initial implant were 1.54 ± 1.67 mIU/mL and 28.2 ± 20 mIU/mL, respectively, in the naïve group, and 0.36 ± 0.33 mIU/mL and 2.09 ± 2.15 mIU/mL, respectively, in the pretreated group. Duration of implant therapy ranged from 1 to 5 years. There were no treatment failures or withdrawals for adverse events.

Baseline pretreatment testing provided valuable adjunct data regarding the possible lack of utility of random LH values in both the diagnosis of CPP and its monitoring. Prior to the initial implant, 7/20 (35%) treatment-naive subjects had prepubertal 0 minute LH levels (< 0.3 mIU/mL) despite peak GnRHa-stimulated LH levels diagnostic of CPP (> 10 mIU/mL), demonstrating the relatively limited sensitivity of a random LH to diagnose CPP. Also, 6/16 (38%) of previously treated patients on depot leuprolide at the time of implant had unstimulated LH levels > 0.3 mIU/mL (range, up to 1.1 mIU/mL) at the baseline visit.

During histrelin treatment, a total of 308 GnRHa stimulation tests were performed in the 36 children. All subjects in both groups maintained suppression of LH levels for the duration of the study based on the primary outcome measure of peak GnRHa-stimulated LH < 4 mIU/mL. The mean of all peak LH levels obtained during the leuprolide stimulation tests while on therapy was 0.62 ± 0.43 mIU/mL (range, 0.06–2.3 mIU/mL). Levels at 30 and 60 minutes were not significantly different from one another. In females, all estradiol levels drawn at stimulation testing were below the *a priori* threshold of 20 pg/mL, and in boys all testosterone levels were below the *a priori* testosterone threshold of 30 ng/dL.

**Figure 1:**
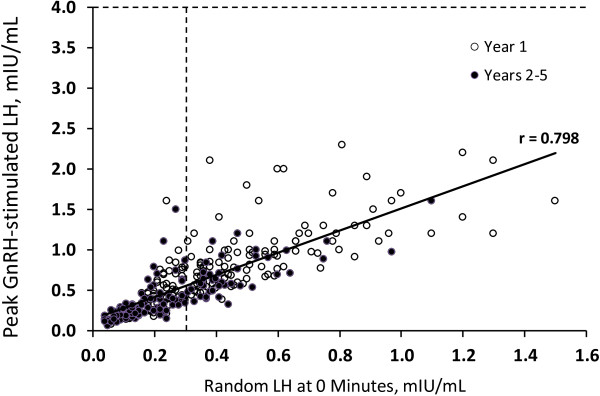
**Scattergram of unstimulated versus peak GnRHa-stimulated LH levels during the histrelin implant therapy.** Data from all follow-up visits (n = 308) during the first year of histrelin implant therapy (open circles) and the second through fifth year of therapy (solid circles) are shown. The horizontal dashed line represents the a priori peak GnRHa-stimulated LH threshold level of 4 mIU/mL defining adequate suppression. The vertical dashed line indicates the published 0.3 mIU/mL pubertal threshold for unstimulated LH levels [15].

The mean of all 0 minute LH levels obtained in leuprolide stimulation tests during therapy was 0.35 ± 0.25 mIU/mL (range, 0.04–1.5). A strong positive correlation was found between the random (0 minute) and peak leuprolide-stimulated LH levels (r = 0.798, Figure 1) (random LH values did not correlate with estradiol or peak FSH). Despite the fact that all peak LH levels were suppressed to < 2.5 IU/L, random LH levels remained at or above the pubertal threshold of 0.3 mIU/mL in 149/308 tests (48.4%) and were ≥ 0.7 mIU/mL in 28/308 (9.1%) and ≥ 1.0 mIU/mL in 7/308 (2.3%). The seven values ≥ 1.0 mIU/mL occurred in 7 different children, and only 4/36 children (11.1%) had random LH < 0.3 at all treatment visits (1/20 naïve, 3/16 previously treated).

**Figure 2:**
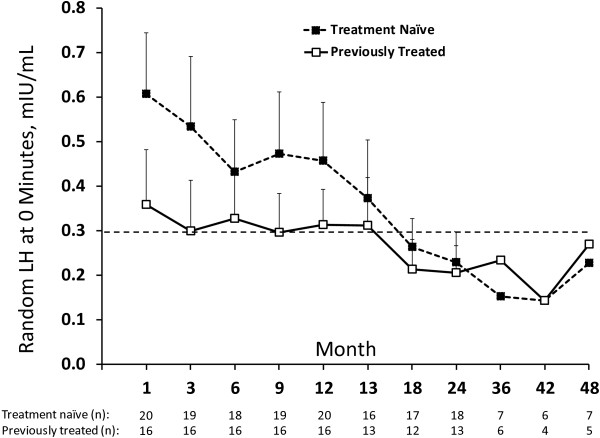
**Mean (± SE) unstimulated LH levels (0 minutes) at each visit over 5 years of histrelin therapy.** The horizontal dashed line indicates the 0.3 mIU/mL pubertal threshold for unstimulated LH levels [15]. Note that the time scale is not proportionate. SE is not shown beginning with the 36-month visit due to small sample size, and values in the fifth year are not shown due to small sample size (3 or less).

A temporal decline in both random and peak LH (Figure 2) was observed over the course of study. Mean random LH levels in the first and subsequent years were 0.42 ± 0.27 mIU/mL (n = 174) and 0.25 ± 0.19 mIU/mL (n = 141), respectively. The highest random LH value was 1.5 mIU/mL during year one, compared with 1.0 mIU/mL during years 2 to 5. Of the 28 basal LH values > 0.7 IU/L during therapy, 24 occurred in the first year. Mean random LH levels were slightly higher in the naïve group compared with the previously treated group during the first year of implant therapy. Six out of 7 random LH values > 1 mIU/mL occurred in the naïve group in the first year of therapy. After the first year, the mean random LH levels in the 2 groups were not different.

## Discussion

All study subjects on histrelin implant therapy for CPP were clinically and biochemically suppressed using the standard outcome measures of peak LH returning to prepubertal levels, reduced sex steroid levels, cessation of advancement of Tanner stage, diminished growth velocity, and reduction in the rate of bone age advancement [6,7]. In this multi-year study of 36 children treated with histrelin implants (20 naïve to treatment and 16 pretreated with another GnRHa), all peak LH values during implant therapy were < 2.5 mIU/mL, demonstrating that puberty in all subjects was unequivocally suppressed. Nonetheless, the random unstimulated LH value exceeded the 0.3 mIU/mL pubertal threshold for the Esoterix assay at 48.4% of the treatment visits, the value was sporadically > 1 mIU/mL in 7 different children, and the lack of suppression assessed by random LH was nearly universal, occurring in 89% of children, despite every other parameter indicating pubertal suppression.

In other words, stimulated LH returns to prepubertal norms during histrelin therapy, but random unstimulated LH levels do not, making the usefulness of random LH in therapeutic monitoring somewhat dubious. The physiologic reason for persistence of basal LH in the pubertal range is not understood. GnRH superagonist therapy might result in tonic LH secretion, but the observed decline over years of therapy would need to be explained. Alternatively, consistently low but measurable LH might be related to the circulating alpha subunits during therapy, although modern assays do not cross-react with those subunits.

Persistence of pubertal random LH values was reported in short-term depot leuprolide therapy nearly 2 decades ago when ultrasensitive LH assays became available [15,16]. It was again recently noted during short-term histrelin therapy [19]. That study was performed with a much smaller sample size, non-simultaneous random and stimulated LH testing, and a relatively insensitive LH assay, but the message is effectively the same. Nonetheless, random LH measurement is commonly performed and used as a criterion to confirm suppression. The persistent elevation in random LH and the temporal decline during the first years of therapy have also been observed during long-term depot leuprolide therapy [2]. It is likely not coincidental that the mean random LH level in the previously treated group in our study was essentially unchanged by subsequent histrelin therapy. Persistent elevation of the random LH level is characteristic of both leuprolide and histrelin therapy, whereas a three-fold reduction in peak LH levels was seen following the change in our pretreated subjects from depot leuprolide to histrelin implant.

Unstimulated LH values trended lower during successive years of histrelin therapy, as did the GnRHa-stimulated LH levels [7]. Our data confirm that random LH levels during GnRHa therapy correlate positively with stimulated LH levels. Nevertheless, a considerable percentage of random LH levels remained at or well above the pubertal threshold in the later years of histrelin treatment. The mean random LH of 0.35 mIU/mL during therapy remained approximately 10-fold higher than the prepubertal mean for this LH ICMA assay, 0.03 ± 0.03 mIU/mL [15], even in the later years of therapy. This extremely low prepubertal norm for random LH using an accurately performed ICMA has been corroborated by other assays [18].

In comparison with the continued elevation of random LH levels, the mean peak LH of 0.62 mIU/mL during therapy in the current study is markedly less than the normal mean prepubertal peak LH (2.0 ± 1.5 mIU/mL) for this ICMA assay [15]. Mean GnRHa-stimulated levels in the later years of therapy fall to near-equivalence with random LH levels. These findings imply that chronic GnRH superagonist therapy results in low-level tonic LH secretion, but nearly complete suppression of pulsatility. As a consequence, only the GnRHa-stimulated LH level during therapy provides clear biochemical confirmation of suppression of the pubertal axis.

During histrelin therapy, assessment of peak LH requires an aqueous leuprolide injection, unlike GnRHa injection therapies in which the therapeutic injection itself can be used as the stimulating agent [4,17,18]. Thus, it is not surprising that some practitioners have been using unstimulated LH levels for convenience or cost savings in monitoring histrelin therapy, along with clinical features and random sex steroid levels. Some utilize 24-hour leuprolide-stimulated estradiol, which circumvents estradiol assay limitations but is likely more inconvenient to obtain than stimulated LH because of the necessity of a return visit. A consensus statement 5 years ago on pediatric uses of GnRHa [1] did not take a position on the utility of random LH for monitoring because the practice was common and published data using sensitive LH assays were at that time limited. Our findings clearly demonstrate that the practice of relying upon random LH for monitoring should be used cautiously, if at all.

## Conclusions

In this study of 36 children, no treatment failures occurred during histrelin implant therapy as assessed by leuprolide stimulation testing and by clinical parameters such as cessation of pubertal progression and diminished growth velocity. Considering that 89% of these subjects exhibited a random LH in the pubertal range at some point during therapy, random LH is unsuitable for routine therapeutic monitoring. A two-step test sequence of random LH followed by as-needed stimulation testing seems futile when failure of the first test is so common. An argument can be made that clinical parameters alone (particularly slowing of growth and decrease in breast size) suffice for treatment monitoring, at least during histrelin therapy. A GnRHa stimulation test with estradiol should be performed if laboratory confirmation is desired, specifically if there are doubts about clinical suppression.

## Abbreviations

CPP: Central precocious puberty; FSH: Follicle-stimulating hormone; GnRHa: Gonadotropin-releasing hormone agonist; ICMA: Immunochemiluminescent assay; LCMS/MS: Liquid chromatography and tandem mass spectrometry; LH: Luteinizing hormone; RIA: Radioimmunoassay; SD: Standard deviation; SE: Standard error.

## Competing interests

EKN, LAS, MEG, and PST have received research support and have been a consultant, advisory board member, or speaker’s bureau member for Endo Pharmaceuticals Inc. EKN, LAS, and MEG have received research support and have been a consultant, advisory board member, or speaker’s bureau member for Abbott. TMD is an employee, and EG was formerly an employee of Endo Pharmaceuticals Inc. and is currently an employee of Synchrony Healthcare, West Chester, PA). Funding was provided by Endo Pharmaceuticals Inc to support editorial assistance in the preparation of the manuscript.

## Authors’ contributions

The authors take full responsibility for all content. The authors verify that they have met all of the journal’s requirements for authorship and that they have not received compensation for this work. All authors were involved in study design and the collection, analysis, and interpretation of data. EKN was primarily responsible for writing the manuscript. All authors have read, approved the final manuscript, and made the decision to submit the manuscript for publication.
